# Photobiomodulation in acute rejection of fetal intestinal grafts: morphological aspects and lymphocyte activation

**DOI:** 10.1007/s10103-026-04883-8

**Published:** 2026-05-11

**Authors:** Sérgio Honório, Priscila Barbosa Carvalho, Regiane Cristine de Souza Almeida, Allan Evangelista Alves Costa, Jacqueline de Fátima Jacysyn, Márcia Kiyomi Koike, Fernando Russo Costa do Bomfim, Edna Frasson de Souza Montero

**Affiliations:** 1 Federal University of São Paulo, São Paulo, Brazil; 2 Universidade de São Paulo, São Paulo, Brazil; 3 Postgraduate Program in Dentistry, Centro Universitário Herminio Ometto de Araras, Araras, Brazil

**Keywords:** Photobiomodulation, Intestinal transplantation, Low-level laser therapy, Immune modulation, Tissue repair

## Abstract

Acute rejection remains a significant challenge in organ transplantation. Photobiomodulation (PBM) has demonstrated anti-inflammatory and regenerative properties, suggesting potential benefits in modulating immune responses. This study aimed to evaluate the effects of PBM on fetal intestinal transplantation in mice.​ The study was conducted in two phases. In the first phase, 77 mice were utilized to assess histological outcomes. Fetal intestines from pregnant C57BL/6 mice were transplanted into BALB/c recipients, forming allogeneic groups (ALLO-GTx) with or without PBM (ALLO-GTxPBM), and syngeneic controls (RG). PBM was applied transcutaneous at the graft site immediately post-surgery (660 nm, 40 J/cm², 1.5 J). Grafts were collected on postoperative days 3, 5, and 7 for hematoxylin-eosin staining and evaluated using Aubert’s criteria. In the second phase, spleens from 30 transplanted mice were analyzed on day 7 via flow cytometry for lymphocyte activation markers (CD4+, CD8+, CD44, CD69, CD54, CD62L).​ PBM significantly reduced rejection scores on days 5 (3.5 [3–9] vs. 12 [10–13.8]; *p* = 0.0006) and 7 (*p* = 0.0096), and enhanced graft development (*p* = 0.0079). Flow cytometry revealed decreased activation of CD4 + CD44+CD69+ (9.08% to 3.15%) and CD8 + CD44+CD69+ (1.53% to 0.90%) T cells in PBM-treated animals. Additionally, lower expression levels of CD62L + and CD54 were observed, indicating immunomodulatory effects. PBM improved graft viability and mitigated acute rejection in fetal intestinal transplantation in mice. These findings support further investigation into PBM as a therapeutic strategy to modulate immune responses in transplantation.

**Supplementary Information:**

The online version contains supplementary material available at 10.1007/s10103-026-04883-8.

## Introduction

The success of solid organ transplantation remains limited by immunological challenges, with graft rejection representing a major obstacle to graft efficacy and longevity. Acute cellular rejection involves an inflammatory response initiated by the recipient’s immune system recognizing the transplanted tissue as foreign, activating cellular and humoral mechanisms that compromise graft viability [1].

Intestinal transplantation, in particular, presents unique immunological characteristics that render it more susceptible to acute and chronic rejection, posing a significant challenge in clinical transplantation. Unlike organs such as the liver, kidney, or heart, the small intestine is characterized by a substantial population of immunocompetent cells and is considered a highly immunogenic organ [2]. As reported in the 2022 OPTN/SRTR report, despite advancements in the field, intestinal graft survival rates at three and five years remain constrained, reaching 26.7% and 42.4%, respectively. Furthermore, a consensus regarding the most efficacious induction immunosuppressive regimen to prevent acute rejection in the first year post-transplantation remains elusive [3].

Currently, standard immunosuppressive protocols involve the administration of tacrolimus in conjunction with corticosteroids. Several monoclonal antibodies are under investigation. Furthermore, innovative strategies to prevent rejection are being explored, including anti-TNF therapy with infliximab, antibodies targeting cell adhesion molecules such as the integrin α4β7 blocker, microbiome manipulation, T-cell therapy utilizing chimeric antigen receptors, and tissue engineering approaches involving stem cell-derived intestinal organoids. The development of effective rejection prevention strategies necessitates a comprehensive understanding of the targeted immune interactions. The modulation of these events depends on the interplay between the donor and recipient immune systems. Regulatory T cells (Tregs), a subset of lymphocytes relevant in the intestine, can be derived from CD4 + or CD8 + progenitors [4].

CD4 + T cells contribute to the inflammatory microenvironment through the secretion of pro-inflammatory cytokines, whereas CD8 + T cells exhibit direct cytotoxic effects, promoting epithelial cell apoptosis via the Fas/FasL pathway. The activation and infiltration of these lymphocyte subpopulations have been strongly correlated with acute intestinal graft rejection, with CD4, CD8, CD44, and CD69 markers serving as critical elements for characterizing this immune response [5].

Photobiomodulation (PBM), a low-power laser electrotherapeutic modality, is recognized for its anti-inflammatory, analgesic, and healing properties, offering a non-invasive alternative to pharmacological interventions with fewer reported adverse effects. In this context, PBM, also referred to as low-intensity laser therapy, has emerged as a promising therapeutic approach. This non-invasive modality exhibits minimal significant adverse effects and employs light within specific parameters (typically 600–1070 nm) to modulate cellular biological processes, thereby inducing anti-inflammatory, analgesic, and tissue repair effects [6].

The absorption of light by mitochondrial chromophores, such as cytochrome c oxidase, initiates a cascade of intracellular signaling events that lead to alterations in gene expression, cell proliferation, and the modulation of inflammatory mediators [7].

PBM as a therapeutic intervention has demonstrated the capacity to reduce the population of inflammatory cells, including macrophages, neutrophils, and lymphocytes, as well as lymphocyte activation [8].

The capacity of PBM to modulate inflammatory mediators and reduce their levels suggests its potential as a therapeutic strategy for controlling the inflammatory response. By restoring the balance between pro- and anti-inflammatory cytokines, PBM therapy exerts a beneficial effect on the acute inflammatory response, contributing to inflammation reduction and immune system regulation [9].

These investigations underscore the influence of PBM on cellular and physiological processes, indicating its therapeutic potential across diverse clinical applications. The mechanistic basis of this phenomenon is closely associated with the interaction of laser light with cellular organelles and their intrinsic biochemical processes. Two key parameters simultaneously influence these effects: wavelength and energy density. The interplay between these two parameters exhibits a continuous relationship [7].

Given the aforementioned evidence, the present study aimed to evaluate the effects of PBM on the morphological aspects of development and acute rejection in murine fetal intestinal transplantation, as well as on lymphocyte activation. This investigation sought to elucidate PBM’s potential as a therapeutic adjunct in the modulation of acute cellular rejection and the enhancement of graft viability.

## Methods

This study was approved by the Animal Ethics Committee of the Federal University of São Paulo, under numbers 6,015,210,722 and 8,870,220,124. The calculation of the appropriate sample size was performed using Altman’s nomogram, as described by Whitley and Ball [10]. Considering a statistical power of 95% and a p-value < 0.01, we used the rejection difference on the 7th DPO between the allogeneic and syngeneic groups from a previous study [11]. The estimated sample size was 4 to 5 animals per group.

**Fig. 1:**
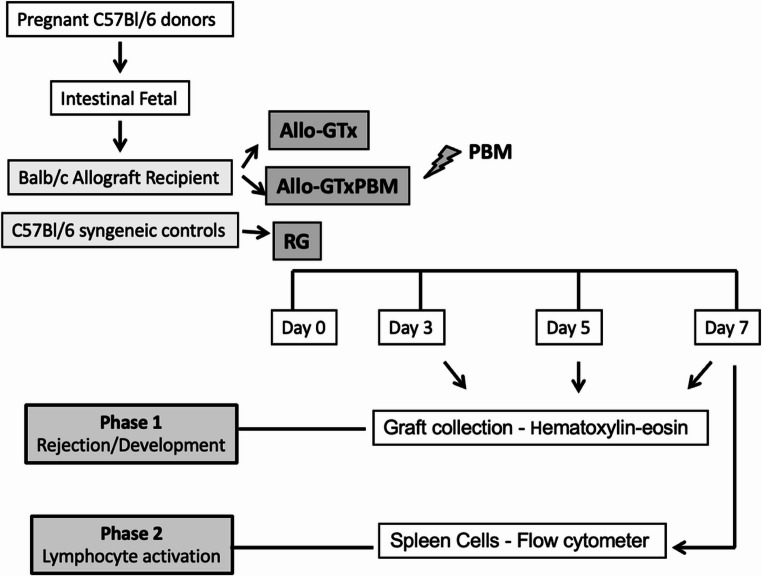
**–** Experimental design: Fetal intestines from pregnant C57BL/6 mice were transplanted into BALB/c recipients, ALLO-GTx, ALLO-GTxPBM, and syngeneic controls (RG). PBM was applied transcutaneous at the graft site post-surgery (Day 0). In the phase1 the grafts were collected on postoperative days 3, 5, and 7 for hematoxylin-eosin staining. In the phase2, spleens were analyzed on day 7 via flow cytometry for lymphocyte activation markers

This study was conducted in two phases to investigate PBM’s effects on fetal intestinal allografts in mice. In Phase 1 (rejection/development), 77 mice were used: 5 pregnant C57BL/6 donors, 48 male BALB/c allograft recipients (ALLO-GTx, *n* = 24; ALLO-GTxPBM, *n* = 24), and 24 male C57BL/6 syngeneic controls (RG). Groups were euthanized on postoperative days 3, 5, and 7 (*n* = 8 for each day). Based on the rejection results on the different days studied, a seven-day postoperative period was chosen for the study of lymphocyte activity in the second phase of the research. The choice was determined by the higher levels of rejection, maintaining the reduction effect by PBM. Phase 2 (lymphocyte activation) utilized 34 mice: 4 pregnant donors, 20 male BALB/c allograft recipients (± PBM), and 10 male C57BL/6 syngeneic controls, all euthanized on day 7. Four-week-old male C57BL/6 and BALB/c mice (24–28 g) were used. Intestinal grafts (three 1 cm segments per fetus) were obtained from C57BL/6 fetuses after maternal euthanasia and fetal excision (Fig. 1).

### Surgical procedures

Anesthesia was induced with intramuscular ketamine (70 mg/kg) and 2% xylazine (10 mg/kg), adjusted as needed. Microsurgery was performed for all procedures. According to the previously described model [11, 12], a maternal laparotomy was performed on female C57BL/6 mice on day 19th of pregnancy to obtain fetal intestines. Using an aseptic technique, a hysterotomy and amniotomy were performed following the laparotomy to expose the fetus (one at a time). Then, a transverse laparotomy was performed on the fetus to exteriorize the small intestine for removal. The harvested intestine was placed in cold saline to clean it of mesentery, then divided into 1-cm segments. The grafts were immediately transplanted into the recipient’s space between the posterior rectus abdominis sheath and the peritoneum. The graft was then inserted into this space. Recipient 5 mm left subcostal incisions were closed with continuous suture 8 − 0 Prolene. Grafts (Phase 1) or spleens (Phase 2) were explanted on designated days post-euthanasia. Euthanasia was by cervical dislocation under anesthesia with ketamine (210 mg/kg) and 2% xylazine (30 mg/kg).

### Photobiomodulation (PBM)

Immediately following the suturing process, the animals in the ALLO-GTxPBM group were performed a single application of PBM on the site where the graft was lodged, situated just above the incision, at a distance of 5 mm from the animal’s skin, with the animals still under anesthesia. The PBM was applied using a calibrated, low-intensity gallium arsenide and aluminum laser device manufactured by D.M.C. Equipamentos LTDA EPP, São Carlos, SP, Brazil, model ImplanTek Lase. The device had a wavelength of λ = 660 nm, nominal power of 30mW, power density of 100mW/cm [2], fluence of 40 J/cm^2^, beam diameter of 0.02 mm, exposure time of 47 s, and an energy of 1.4 J at a single point [13–16].

## Sample processing

### Histological analysis of graft development and rejection

Grafts were fixed, processed, sectioned, and stained with hematoxylin-eosin (code 51275 and 318906, Sigma-Aldrich, Merck KGaA, Darmstadt, Germany) at UNIFESP Pathology laboratory. Development and rejection were scored (0–13 and 0–22, respectively) using [11] criteria, which establish two different scores, containing five criteria for evaluating development and four for rejection. The development criteria involve analysis of the presence of digestive epithelium, the importance of mucous secretion, the development of the muscle layer, the development of crypts, and the development of villi. The rejection criteria evaluate the characteristics of the cellular infiltrate, the importance of the peri- and subcrypt infiltrate, the importance of the muscular infiltrate, and the damage to the crypts and villi. These criteria receive different scores that determine the final score for graft development or rejection.

### Lymphocyte activation analysis

Spleens were harvested, homogenized in PBS, and lymphocytes isolated by centrifugation and RBC lysis. 1 × 10⁶ cells were labeled with fluorochrome-conjugated antibodies (CD3, CD4, CD8, CD62L, CD54, CD69, CD44; Pharmingen, BD, San Jose, CA, USA) and analyzed using a FACSCanto II flow cytometer (100,000 events, BD, San Jose, CA, USA) and FlowJo software (Ashland, OR, USA).

### Statistical analysis

Non-parametric tests (Mann-Whitney for intergroup comparisons) analyzed data, presented as medians with interquartile ranges. Significance was set at *p* < 0.05. For the analysis of lymphocyte activation molecules, normality was assessed, and group comparisons between the Control and PBM conditions were performed using parametric (T-test) or non-parametric (Mann-Whitney U test) statistics, as determined by the normality of the data.

## Results

### Histological analysis

**Fig. 2:**
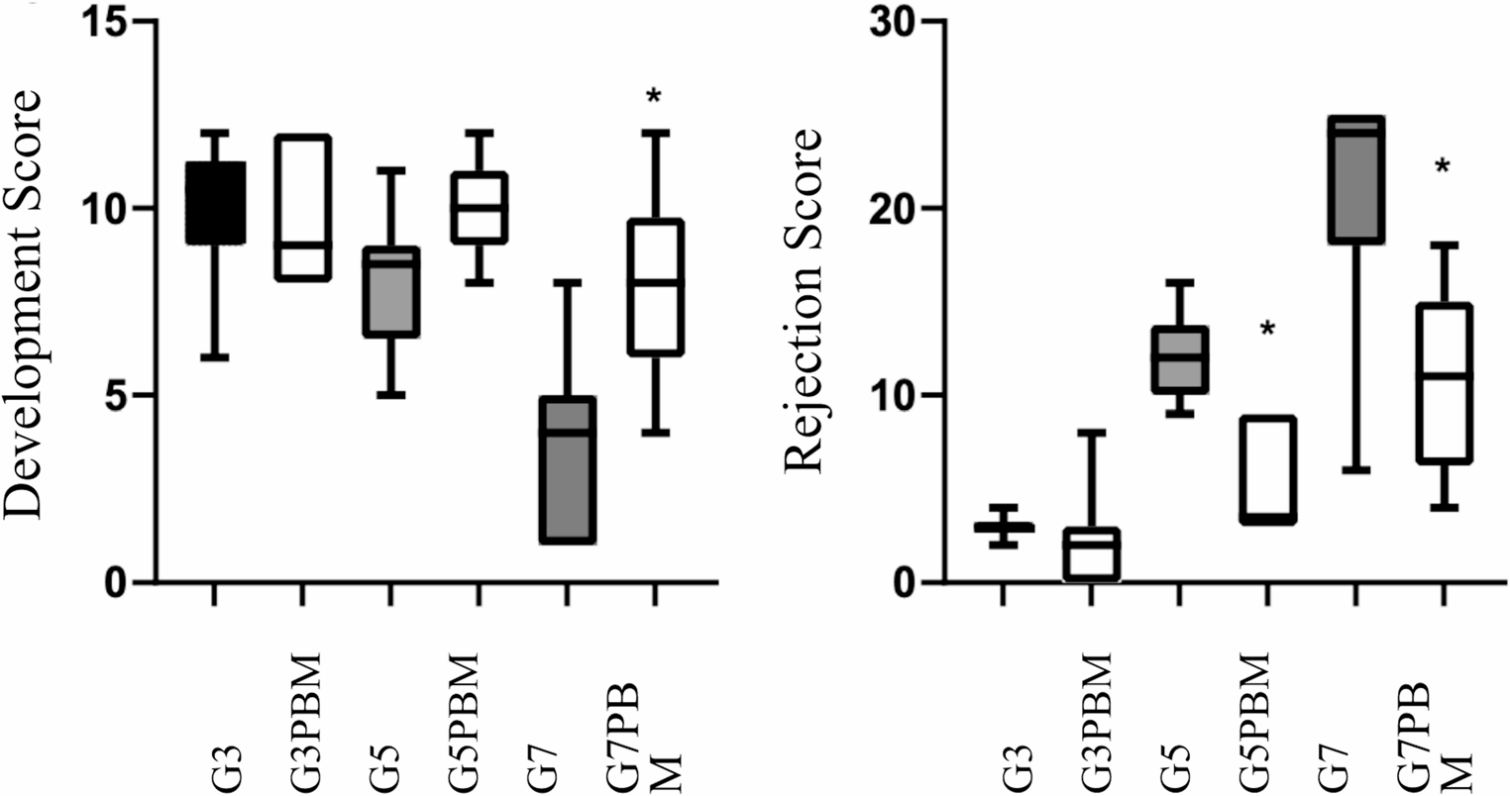
Graft development and rejection scores in groups with and without PBM on postoperative days 3, 5, and 7. Mann-Whitney intergroup test; *, *p* < 0.05

The intestinal grafts of syngeneic animals demonstrated a progressive enhancement in development scores over time following transplantation. Consequently, an elevated development score is observed from the third postoperative day onwards, persisting until the seventh day (11, 11, and 13, respectively). A slight increase in the rejection score was observed in the periods evaluated, 3rd, 5th, and 7th, reaching 2.0, 4.7, and 5.7, respectively, with inflammatory infiltrate and neovascularization (Table 1; Fig. 2).

The allogeneic groups demonstrated a highly significant inflammatory infiltrate, in addition to villi and intestinal epithelium destruction. The development of intestinal grafts was sustained until the 5th day of the experiment, with the application of PBM. However, on the 7th day, grafts from both groups, with and without PBM, exhibited a significant decrease in the degree of development. Nonetheless, PBM was able to maintain superior levels. In the rejection assessment, there was an intensification of the inflammatory process, reaching scores close to the maximum possible score in grafts without PBM on the 7th postoperative day. However, PBM was found to attenuate the acute rejection process, maintaining the score at a level 55% lower than that which would be expected in the absence of intervention (Table 1; Fig. 2).

**Table 1:** Graft development and rejection scores in allogeneic groups with or without PBM, according to Aubert et al. [11] criteria

POD	Development			Rejection		
	ALLO-GTx (*n* = 6–8)	ALLO-GTxPBM (*n* = 7–8)	*P* Value	ALLO-GTx (*n* = 6–8)	ALLO-GTxPBM (*n* = 7–8)	*P* Value
3	10.5 (9–11.3.3)	9 (8–12)	0.9767	3 (2.8–3.3)	2 (0–3)	0.1148
5	8.5 (6.5–9.5)	10 (9–11)	0.0665	12 (10–13.8.8)	3,5 (3–9)	0.0006^*^
7	4 (1–5)	8 (6–9.8.8)	0.0079^*^	24 (18–25)	11 (6.3–15)	0.0096^*^

#### **Statistical analysis**

**Fig. 3:**
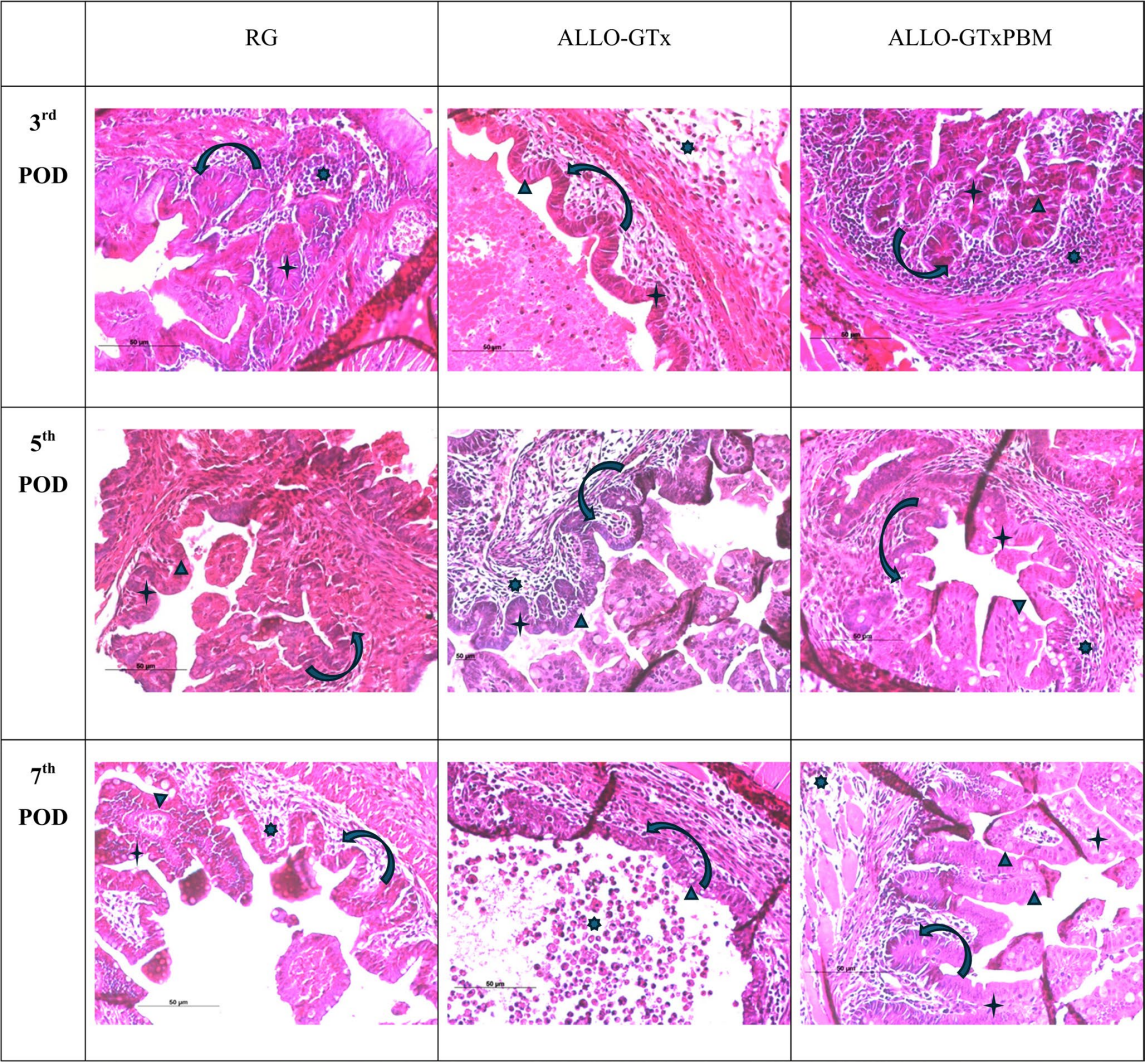
Photomicrograph of intestinal grafts stained with HE on different postoperative days. - inflammatory cells.  – villi.  – Goblet cells (mucous secretion).  – Intestinal epithelium. Scale bar - 50µM. The PBM effect is an important distinction between allogenic grafts (ALLO-GTx and ALLO-GTxPBM) on the 7th POD. It lessens inflammatory infiltrate and promotes the development of structured villi containing mucous-producing Goblet cells

Mann-Whitney test; median data (interquartile range); * *p* < 0.05. ALLO-GTx and ALLO-GTxPBM: allogeneic groups with and without photobiomodulation. DPO: postoperative days (Fig. 3).

### Lymphocyte activation analysis

**Fig. 4:**
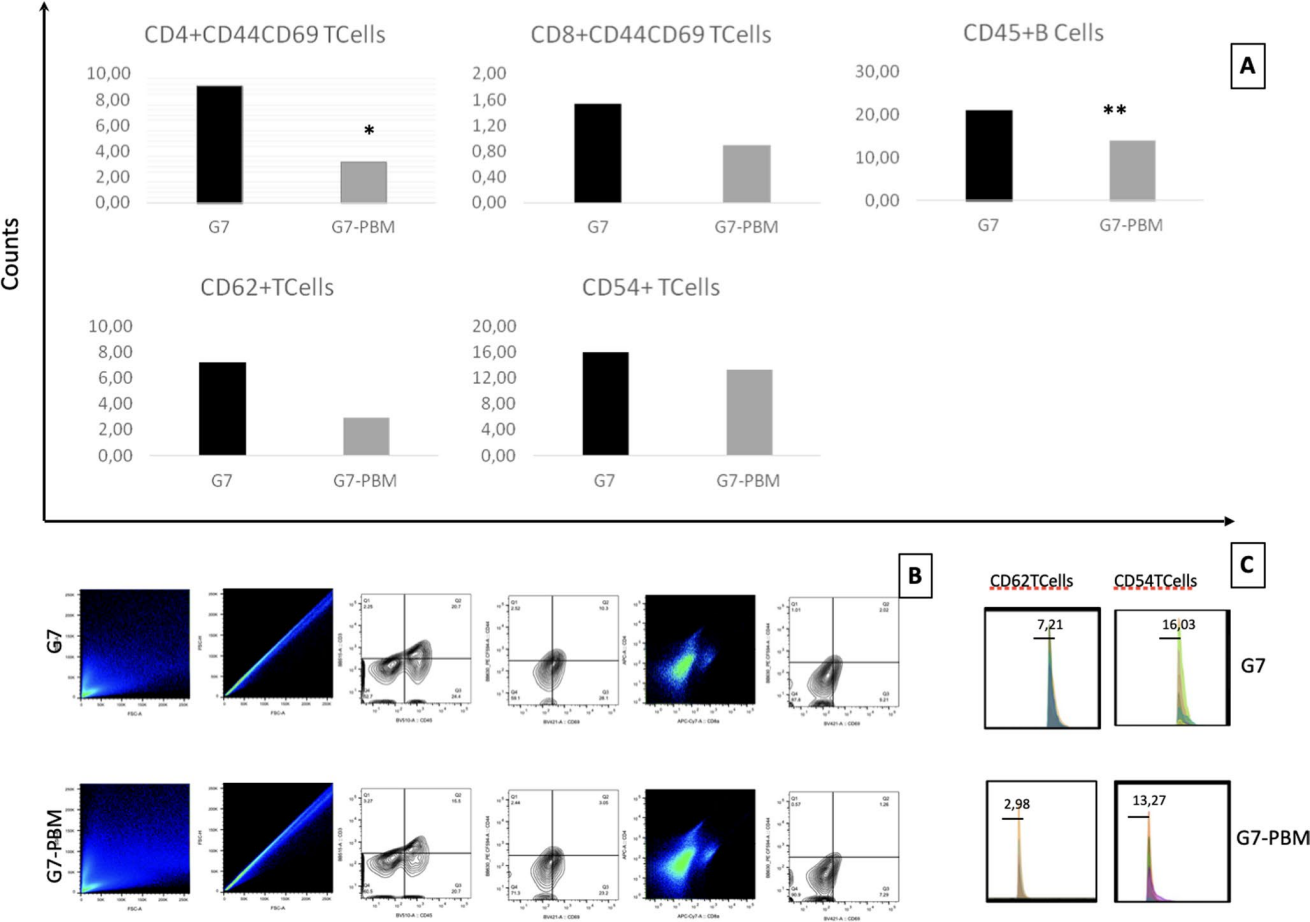
Lymphocyte activation in mice from groups G7 and G7-PBM subjected to allogeneic intestinal transplantation, treated or not treated with photobiomodulation. After 7 days, the animals in groups G7 and G7-PBM were euthanized, their spleens were collected, and the lymphocytes were evaluated for activation molecules by flow cytometry. (**A**) Graph representing the mean values of cells positively stained for fluorescent antibodies for flow cytometry. (**B**) Schematic illustration of the flow cytometry analysis strategy for spleen cells from groups G7 and G7-PBM; (**C**) Representative histogram with the mean values of cells positively stained for CD54 + and CD62L+ antibodies in the evaluated groups. Statistical analysis performed by Student’s t-test (*) and Mann-Whitney test (**), respectively. *P* < 0.05

In evaluating the expression of activation molecules in splenic T lymphocytes of mice in the 7th POD of intestinal allotransplantation, treated or not treated with PBM, a decrease in the activation of TCD4 + CD44+CD69 + cells from 9.08% to 3.15% (*p* = 0.0208) and in TCD8 + CD44+CD69 + cells from 1.53% to 0.90% (*p* = 0.1478) was observed in the ALLO-GTxPBM animal groups when compared to the untreated ALLO-GTx group. Concerning CD3 + CD45+B cells, a lower percentage of these cells was observed in the ALLO-GTxPBM animal groups in comparison to the ALLO-GTx animal group (21.00% to 14.04%; *p* = 0.0043). The ALLO-GTxPBM group demonstrated a reduced expression of both CD62L+ (2.8%) in comparison to the ALLO-GTx group (7.9%) and CD54 expression (13.27%) in relation to the ALLO-GTx group (16.03%) (Fig. 4).

## Discussion

The present study demonstrated that the application of PBM in the context of fetal intestine transplantation in mice was able to modulate the inflammatory response and acute cell rejection, promoting better graft development and less inflammatory infiltrate, especially on the seventh postoperative day. The morphological findings of development and rejection were corroborated by changes in the flow cytometry of lymphocyte activation molecules, such as CD4 + 44+69+, CD8 + 44+69+, and CD62L, which were attenuated with PBM.

Histological analysis, based on [11] criteria, revealed a significant difference in graft development between PBM-treated and untreated groups. On the seventh day, PBM-treated allografts (ALLO-GTxPBM) exhibited a higher development score compared to untreated allografts (ALLO-GTx), approaching scores observed in syngeneic grafts. This suggests that PBM not only attenuates rejection but also fosters a more permissive microenvironment for intestinal graft maturation, potentially by enhancing tissue repair and regeneration. In the present study, PBM reduced the morphological aspects of acute rejection, as inflammatory infiltrate, villi and cripts injury, and intestinal epithelium destruction. Untreated allografts exhibited a progressive increase in rejection scores, consistent with the natural course of allogeneic immune responses characterized by lymphocytic infiltration and tissue damage. In contrast, PBM significantly decreased acute cellular rejection scores, indicating an immunomodulatory effect on the graft. While studies directly evaluating organ transplant are limited, [17] reported that PBM enhanced skin graft viability and integration in rats. Concerning to immunohistochemistry evaluation of IL-4, it was similar in the studied groups. The authors justified this finding by a short time of evaluation and level of energy provided by the PBM parameters. In a systematic review of animal models, [18] selects articles that describe the ability of PBM to improve tissue repair, improve burn healing, mitigate muscle injuries by preventing the increase of oxidative stress proteins, and improve functional recovery and tactile sensitivity in spinal cord injuries. This review details different methods of PBM application, animals, and diseases where it can be used. Furthermore, [19] demonstrated that red light irradiation in human fibroblasts modulates the expression of genes involved in cell proliferation, oxidative stress control, mitochondrial energy metabolism, cytoskeleton and extracellular matrix proteins, and immune/inflammatory/cytokine pathways.

Lymphocyte activation in this intestinal transplant model corroborated the morphological findings. PBM treatment reduced CD4 + and CD8 + T lymphocyte activation, as evidenced by decreased expression of the activation markers CD44, CD69, and CD62L. Jiang and Fan [20] conducted a review of the immunoregulatory mechanisms of CD8 + T lymphocytes, emphasizing the relevance of the CD8Treg to allogeneic transplantation. While CD8 + T cells are known for their role in cell-mediated cytotoxicity and allograft recognition, they also possess immunoregulatory potential. Gonzalez et al. [21] demonstrated that PBM modulated CD8 + cell population during wound healing in rats, with an early decrease followed by a later increase concomitant with a decrease in CD68 + cells, suggesting a regulatory role for CD8 + lymphocytes in reducing inflammation. As Britto et al. [6] found, PBM reduced CD4 + and CD8 + lymphocytes in a murine model of chronic obstructive pulmonary disease. The effect of PBM in this model was analogous to the findings of a negative regulatory effect of PBM on both CD4 + and CD8 + lymphocytes in our transplant model.

The reduced frequency of CD3 + CD45+ cells in PBM-treated animals further supports the hypothesis of decreased activation of adaptive immune cells to the graft site. Notably, PBM also reduced the expression of leukocyte migration and adhesion molecules, CD62L and CD54, potentially contributing to reduced inflammatory infiltration and rejection. Numerous studies support these findings, indicating that PBM modulates multiple levels of the immune cascade, affecting both immune cell activation and migration. Tomazzoni et al. [8] reported that PBM modulated inflammatory mediators in chronic nonspecific low back pain. Neves et al. [9] demonstrated PBM’s capacity to modulate local and spinal cytokine release during inflammatory hyperalgesia in mice. Gonçalves et al. [22] showed that PBM treatment in rats with acute rheumatoid arthritis reduced inflammatory cell influx, pro-inflammatory cytokine production, and metalloproteinase activity in the synovial region, which contributes to synovial fluid and articular cartilage degradation. Abidi et al. [23] observed that PBM negatively regulated inflammatory mediators in stimulated human fibroblasts, suggesting its potential for managing periodontal inflammation.

PBM parameters, such as wavelength, energy dose, and exposure time, can be adjusted to modulate therapeutic effects. Heiskanen and Hamblin [24] presented tables summarizing PBM applications with varying dosages and light types, demonstrating the broad range of potential applications. The range of these protocols is extensive, extending from single applications in rheumatoid arthritis [23] to varied dosages and complementary wavelengths. This extensive range underscores the challenge in defining the optimal protocol for each disease. Abidi et al. [23] observed cytotoxic effects of 660 nm and 810 nm laser wavelengths, and expanded the study to include 980 nm, 660 nm + 810 nm, and 810 nm + 980 nm. Martignago et al. [25] evaluated the in vivo response of different wavelengths (red and infrared) of light-emitting diodes (LEDs) in full-thickness skin grafts in rats. Skin grafts were irradiated with red (630 nm) or near-infrared (850 nm) LEDs. Most studies indicate that wavelengths between 660 nm and 980 nm have effective anti-inflammatory properties, while those between 600 nm and 1070 nm significantly influence cell repair. Abidi et al. [23] reported that 810 nm exhibited anti-inflammatory effects alone and in combination with 660 nm and/or 980 nm. Rola et al.⁷ demonstrated that wavelengths between 600 nm and 1070 nm promote cell proliferation, likely due to reduced light absorption beyond this range, as shorter wavelengths are absorbed by hemoglobin and longer wavelengths by water.

The selection of the 660 nm wavelength was informed by its well-established interaction with mitochondrial chromophores, particularly cytochrome c oxidase, which has been demonstrated to promote ATP production, cell proliferation, and modulation of inflammatory responses [7, 18, 19, 26]. Whilst it is evident that longer wavelengths (> 900 nm) may facilitate deeper tissue penetration, red light has been repeatedly associated with cellular activation and tissue repair processes, particularly in superficial tissues. This supports its utilisation in this experimental model, given the preperitoneal location of the graft [7, 15, 16].

The dosimetry used in this study (660 nm, 30 mW, 100 mW/cm², 40 J/cm², 47 s, 1.4 J per point) was based on literature recommendations for cell modulation without inducing thermal necrosis [13–16]. The effects described in relation to lasers are not observed in this case, based on the findings of Mathioudaki et al. [13]. The study’s intensity range did not elicit any significant thermal effects during its application, which was conducted at a distance of 5 millimeters from the surgical site. The induction of light-induced stress was prevented by ensuring that the animals were under anaesthesia. Gonçalves et al. [22] demonstrated the anti-inflammatory, analgesic, and healing properties of PBM in rats with acute rheumatoid arthritis, highlighting its potential as a non-invasive alternative to pharmacological interventions. Low-level laser irradiation activates indirect mitochondrial pathways, leading to physiological effects such as increased vasodilation without thermal injury, improved local cell tropism, altered gene expression, modulation of pro-inflammatory mediators, and accelerated healing. Freitas and Hamblin [26] reviewed PBM mechanisms, demonstrating that the treatment does not cause significant tissue temperature increases or gross structural changes, distinguishing it from ablative or heating-based light therapies.

It is worth noting that although the device has a nominal beam diameter of 0.02 mm at the emission source, the irradiation was applied at a distance of 5 mm from the skin, thereby enabling beam divergence and effective enlargement of the irradiated area. In addition, the phenomenon of tissue scattering, together with photon diffusion, facilitates a wider distribution of energy in the graft region, even in cases where a single-point application is used, as previously outlined in photobiomodulation studies evaluating light propagation and the optical properties of tissues, especially in biological tissues with heterogeneous optical properties [7, 18, 24, 26].

The present study is subject to certain limitations. Firstly, the restriction of immune phenotyping to the spleen precludes a direct assessment of lymphocytic infiltration within the graft microenvironment. Although graft development/rejection was graded according to the Aubert et al. [11] criteria, it was not done enzymatic digestion of graft tissue to recover graft-infiltrating leukocytes for flow cytometry, primarily due to limited graft size and the need to prioritize morphologic assessment. An integrated approach would facilitate direct correlation of local immune infiltration with histology and treatment response.

A challenge in PBM research is the lack of standardized protocols. The diverse range of techniques and modalities, including low-power lasers, LEDs, and monochromatic or polychromatic light sources, makes PBM a versatile therapy for various medical procedures, including transplantation. Heiskanen & Hamblin [24] reviewed numerous medical conditions (and their animal models) where PBM has been investigated in murine, rabbits, and humans, including diseases affecting the brain, bone, eye, internal organs, connective tissue, skin, muscle, and transplant tissue. Fekrazad & Fekrazad [27] highlighted the potential of regenerative medicine, combining PBM and stem cell therapy, to achieve rapid recovery and reduce organ transplant rejection risk.

The potential of PBM as an adjuvant therapy to mitigate acute rejection and enhance tissue repair in intestinal transplantation, a challenging procedure due to vigorous local immune activity and resident microbiota [2], showed translational promise for PBM in minimally invasive immunosuppression protocols.

## Conclusion

The present study demonstrates that photobiomodulation (PBM) significantly improves graft development and attenuates the morphological features of acute rejection in fetal intestinal transplantation in mice. These histological findings were corroborated by immunological data, which demonstrated reduced activation of key T lymphocyte populations (CD4 + and CD8+) and decreased expression of activation and migration markers such as CD44, CD69, CD62L, and CD54. Taken together, these results suggest that PBM exerts a modulatory effect on both local tissue response and systemic immune activation, thereby contributing to a more favorable microenvironment for graft survival.

From a translational point of view, PBM represents a promising non-invasive adjuvant strategy for modulating immune responses in transplants, with the potential to reduce the doses of immunosuppressive pharmacological agents. It is recommended that subsequent studies investigate the optimisation of PBM parameters, the use of different wavelengths, the frequency of repeated applications, and the combination of immunological analyses at the graft. This will contribute to the further elucidation of the mechanisms and clinical applicability of the treatment.

## Supplementary Information

Below is the link to the electronic supplementary material.

Supplementary Material 1

Supplementary Material 2

Supplementary Material 3

Supplementary Material 4

## Data Availability

Data is provided within the manuscript and supplementary information files.
