# Supplementary material for: Photobiomodulation in acute rejection of fetal intestinal grafts: morphological aspects and lymphocyte activation
Source: Lasers Med Sci. 2026 May 11;41(1):90. doi: 10.1007/s10103-026-04883-8 (PMC13161243; doi:10.1007/s10103-026-04883-8)

## Slide 1
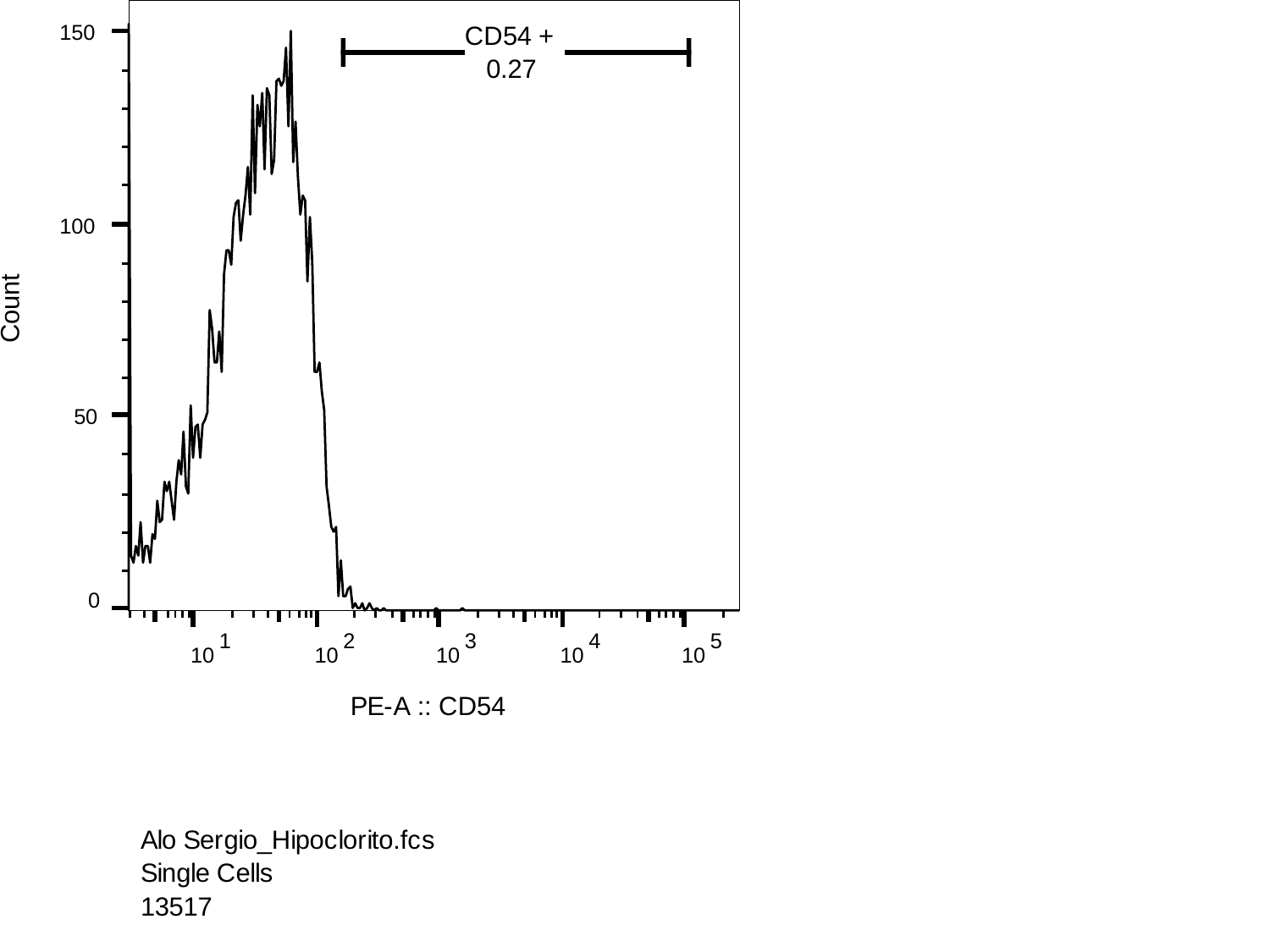

## Slide 2
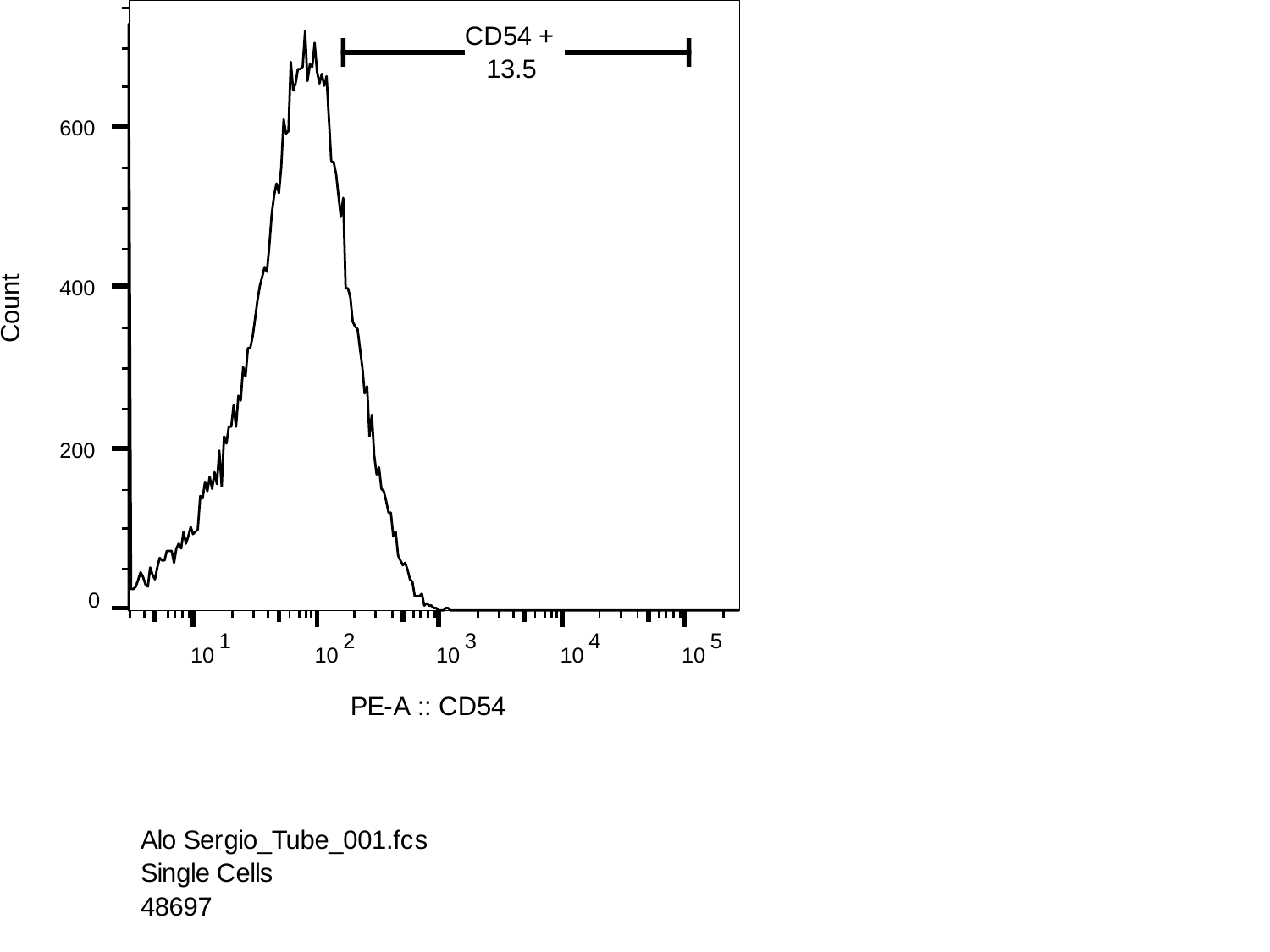

## Slide 3
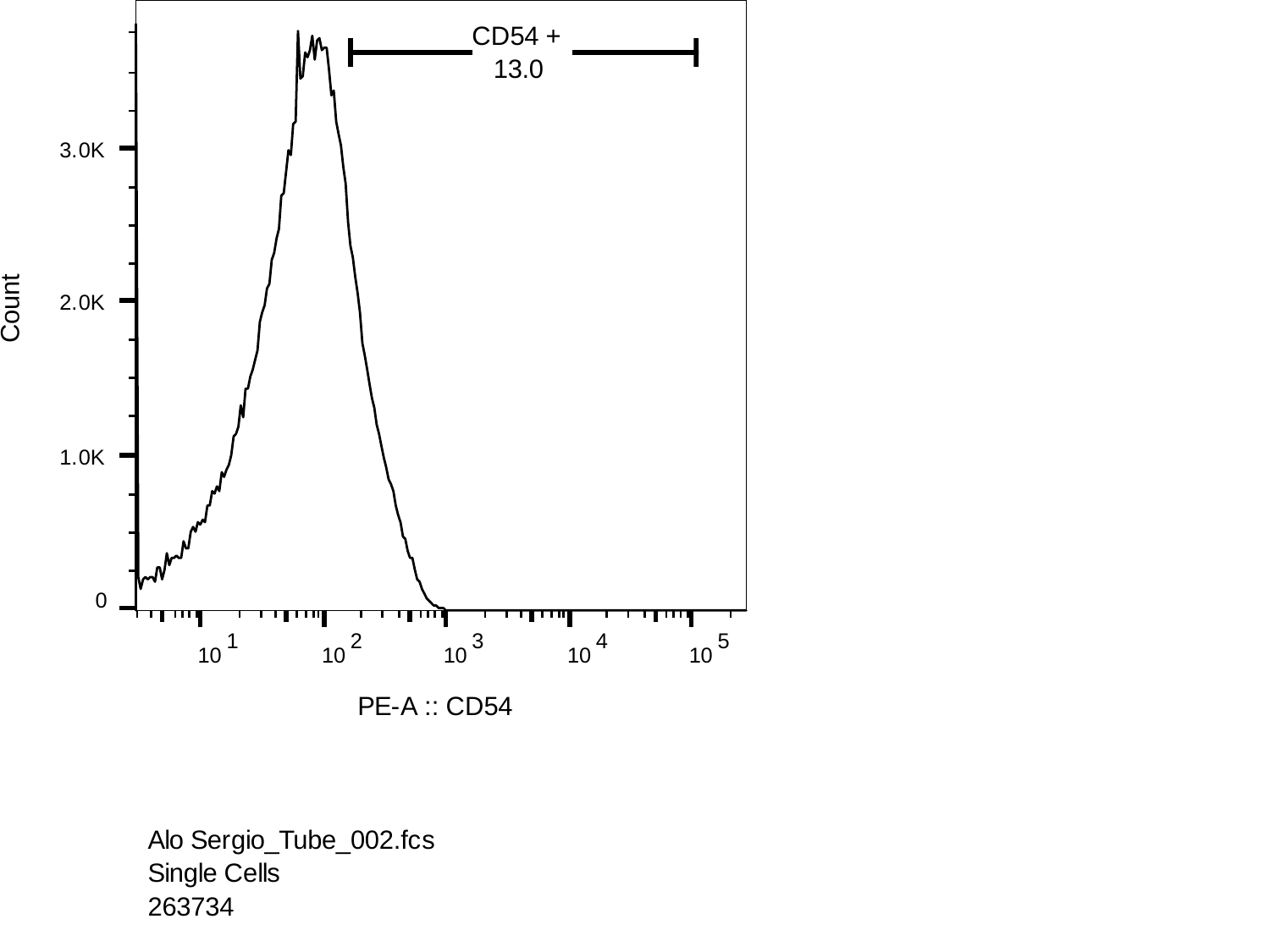

## Slide 4
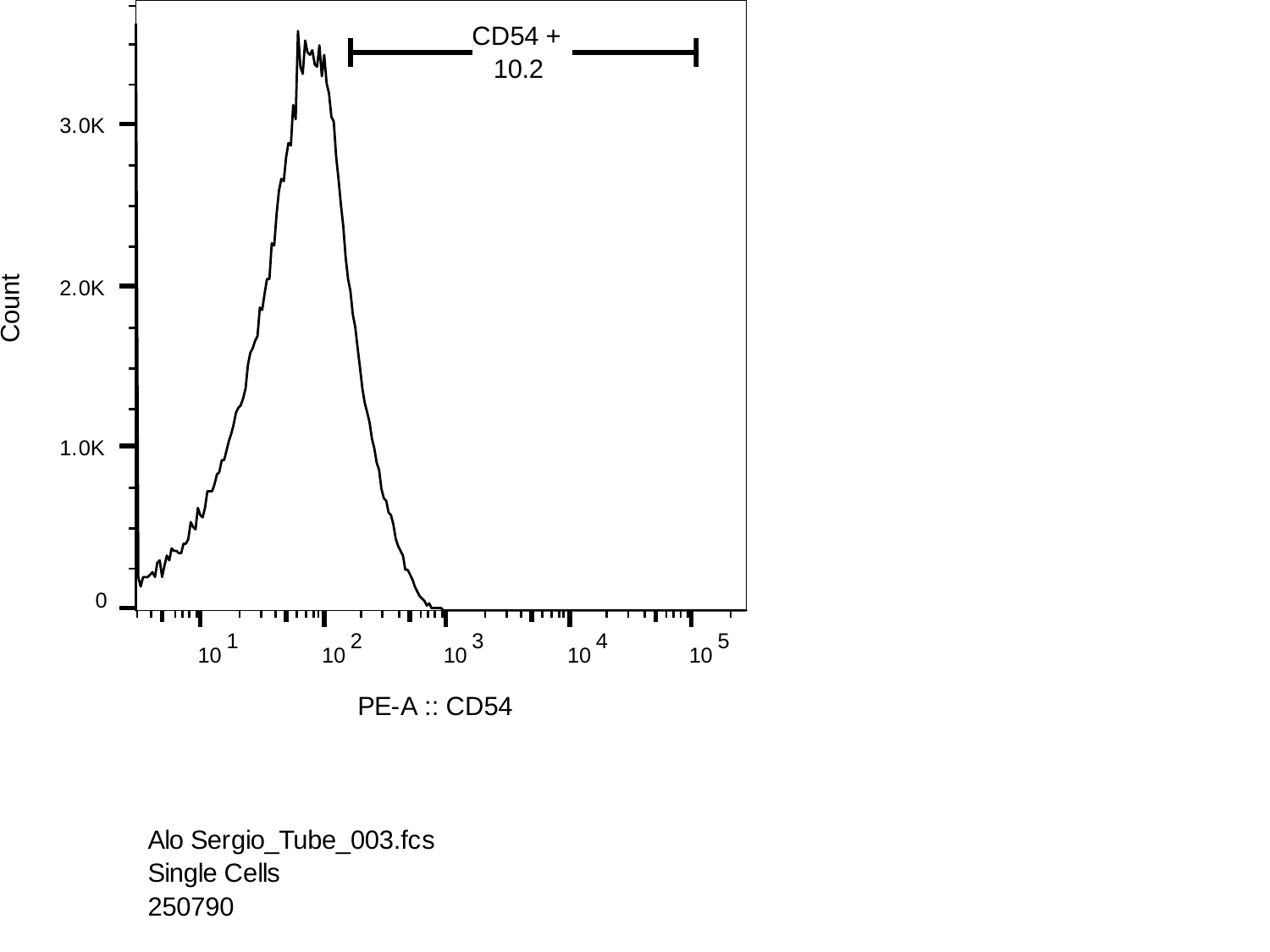

## Slide 5
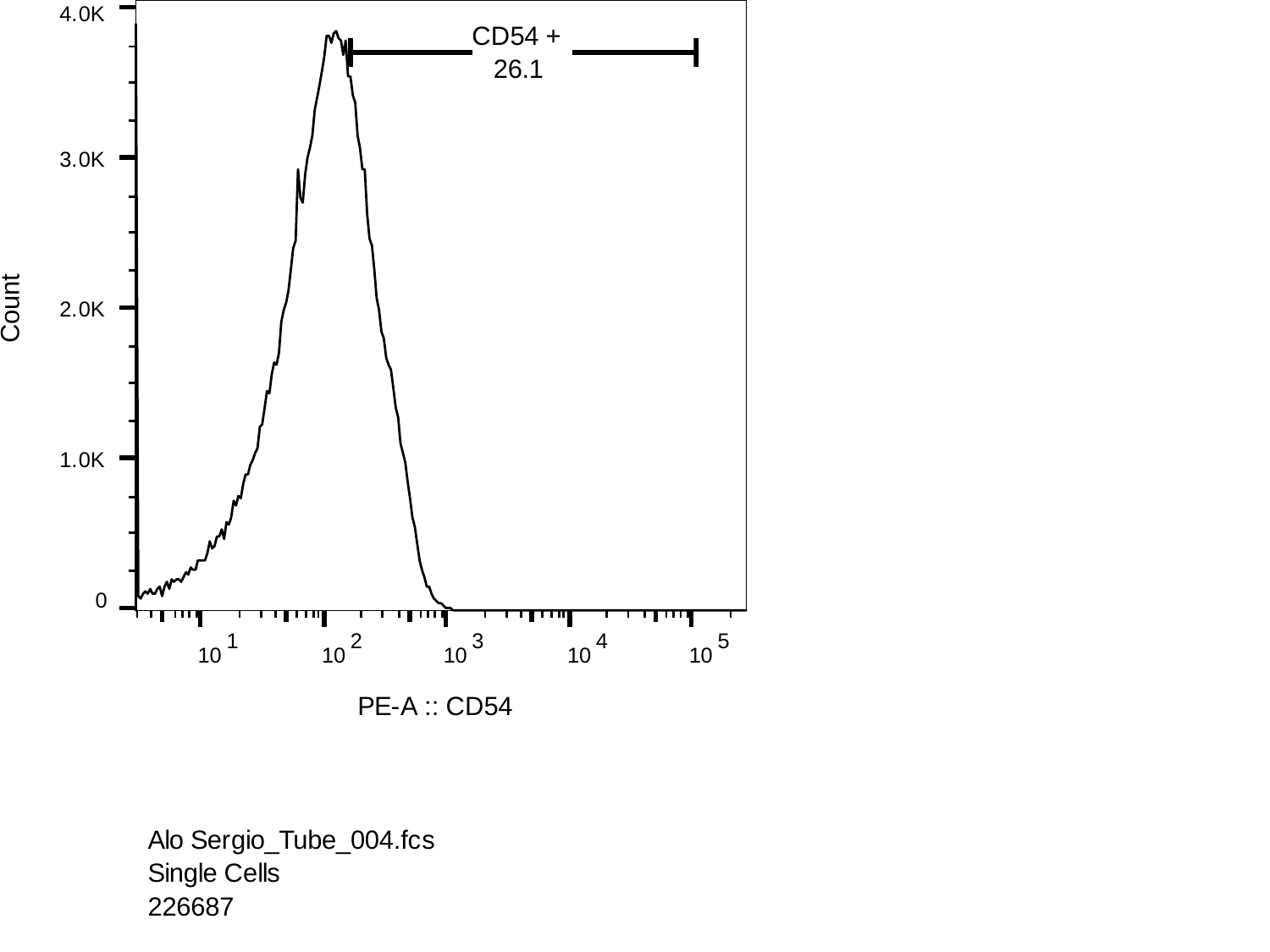

## Slide 6
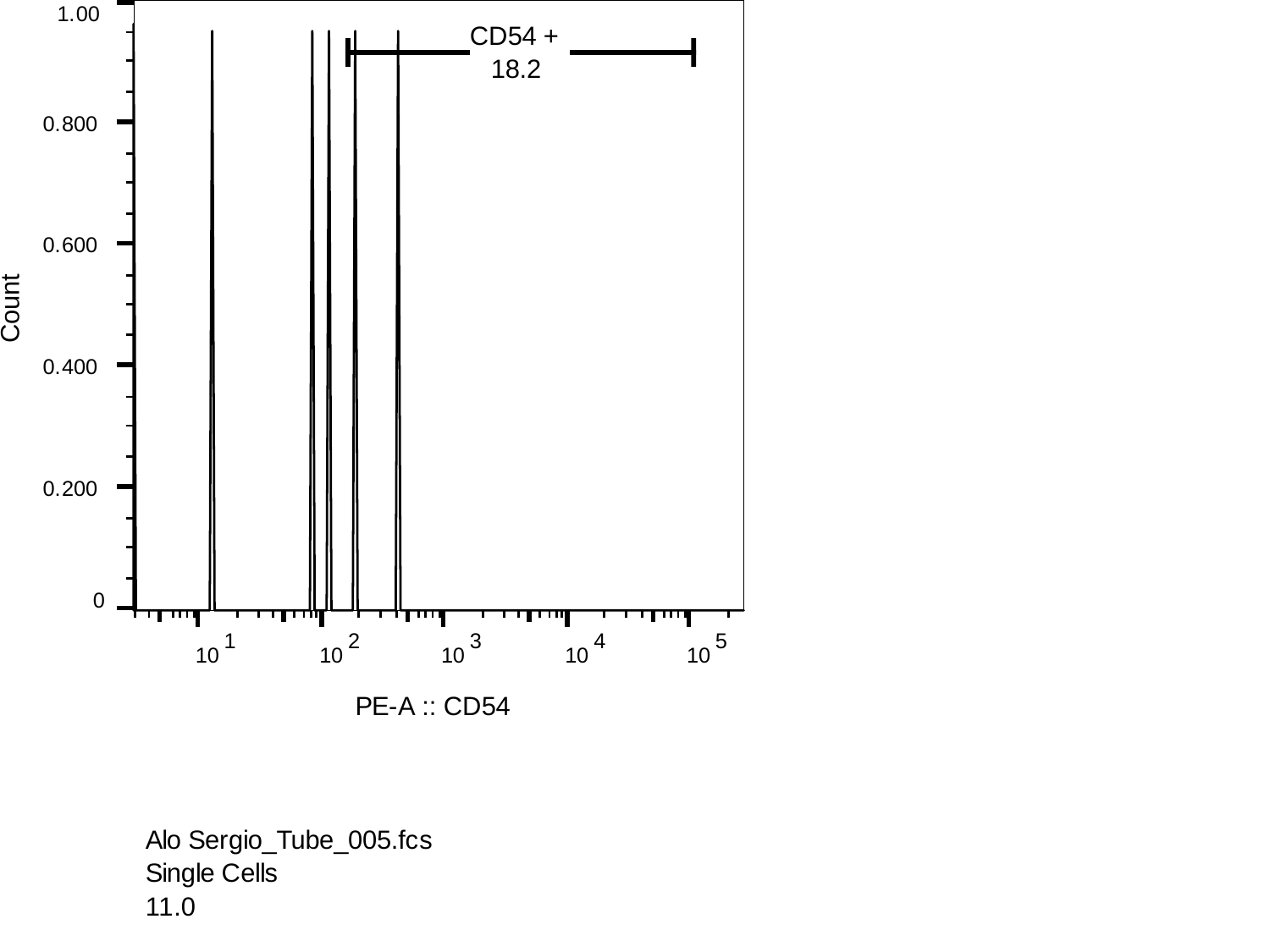

## Slide 7
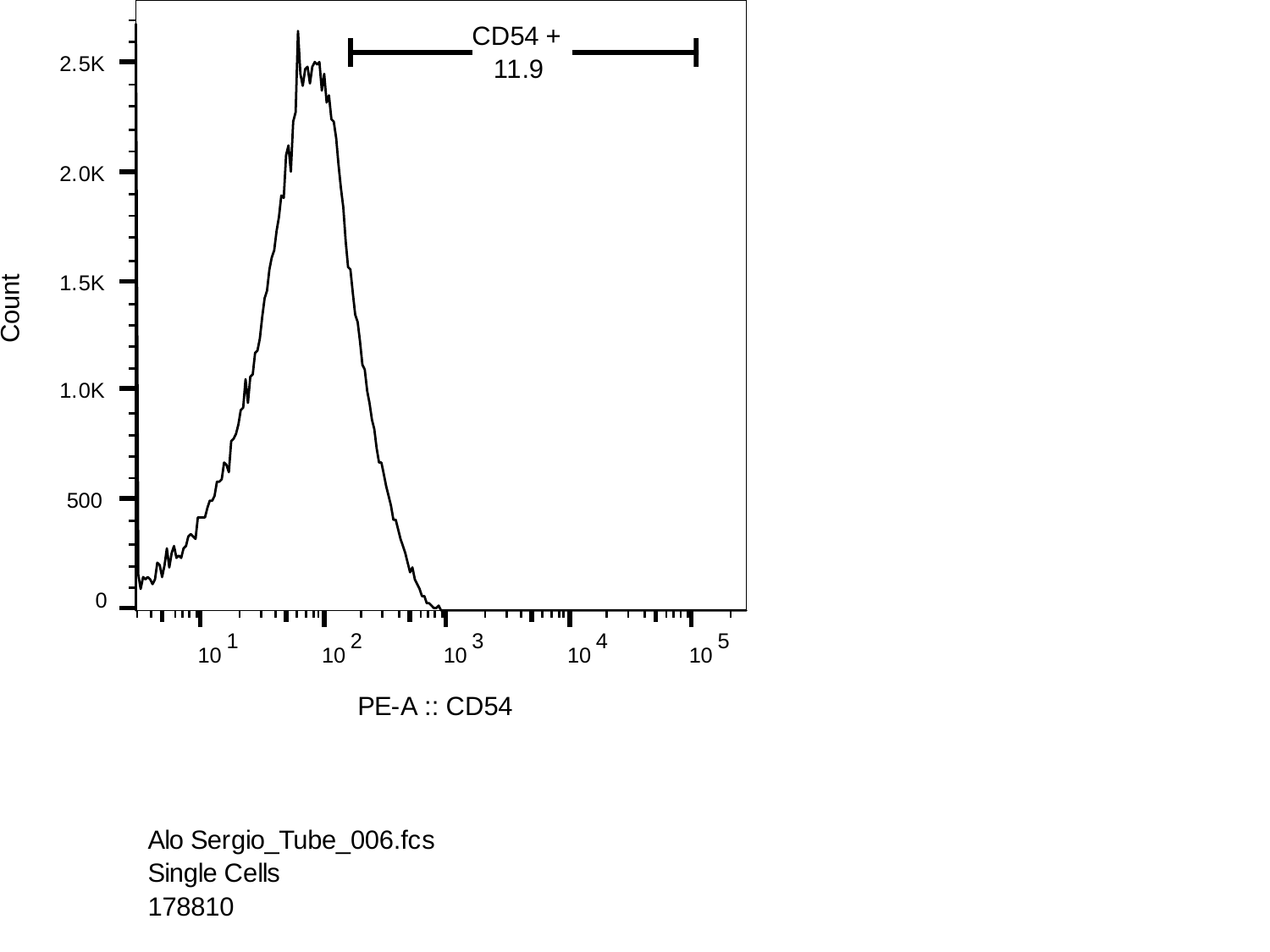

## Slide 8
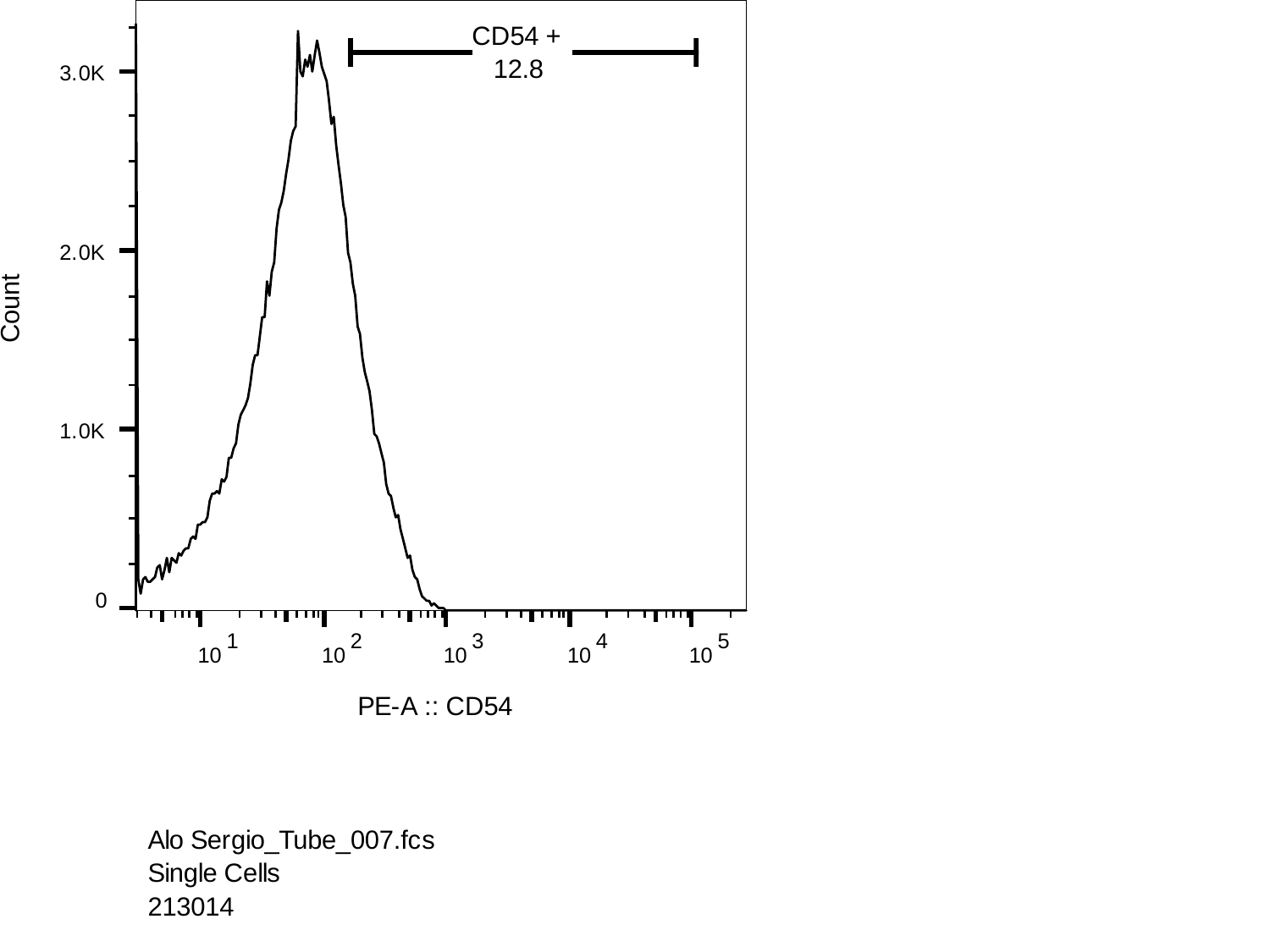

## Slide 9
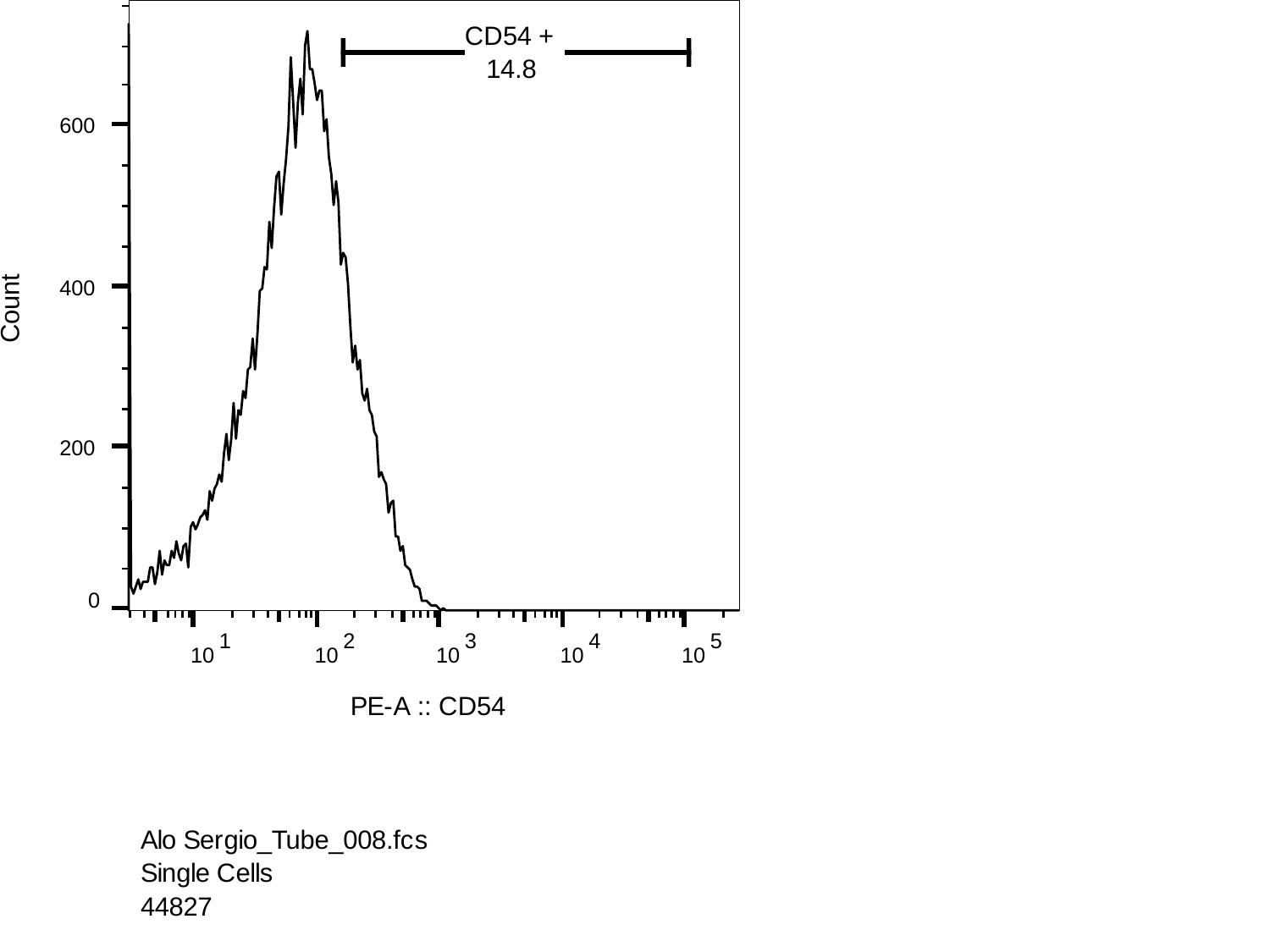

## Slide 10
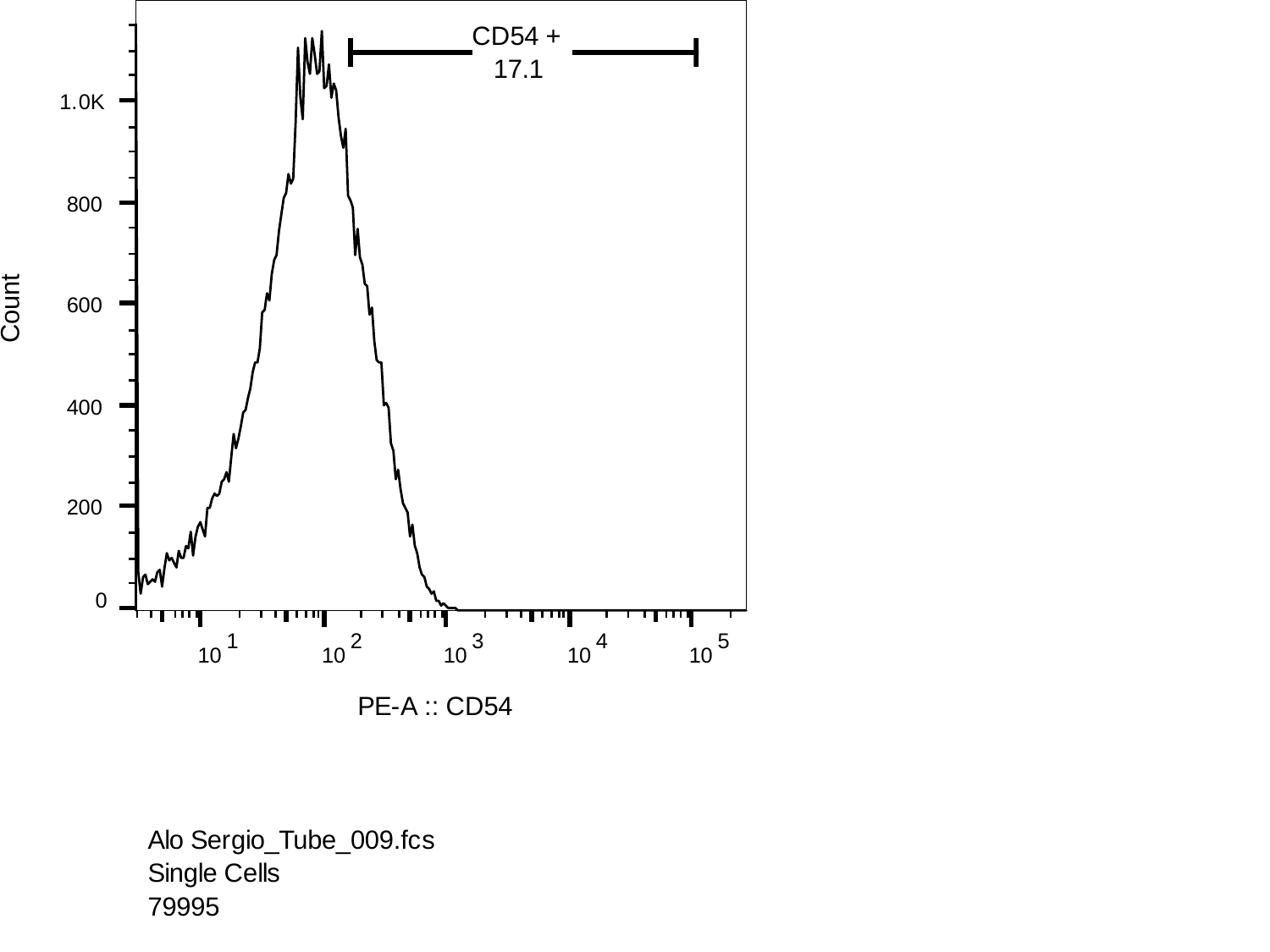

## Slide 11
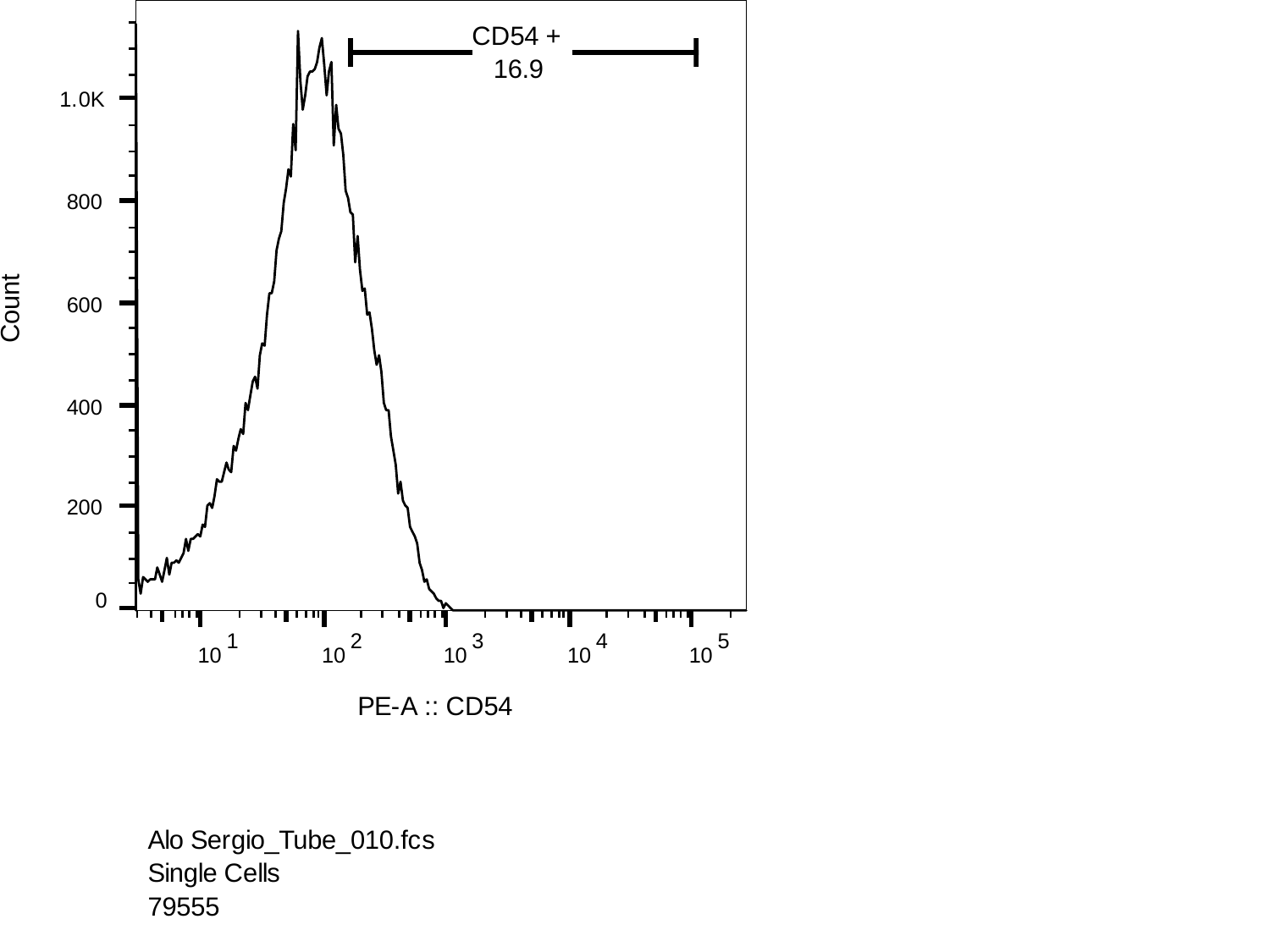

## Slide 12
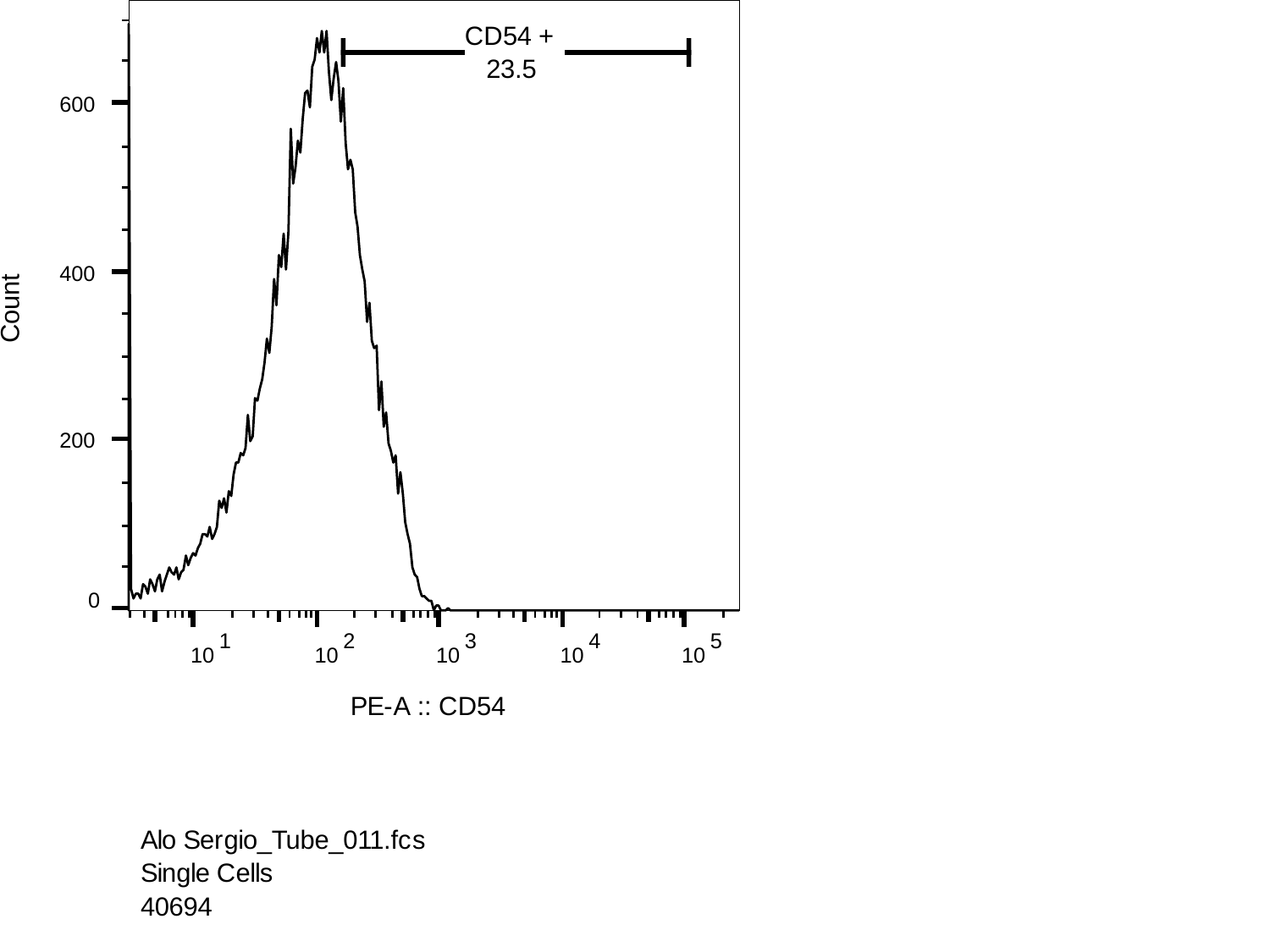

## Slide 13
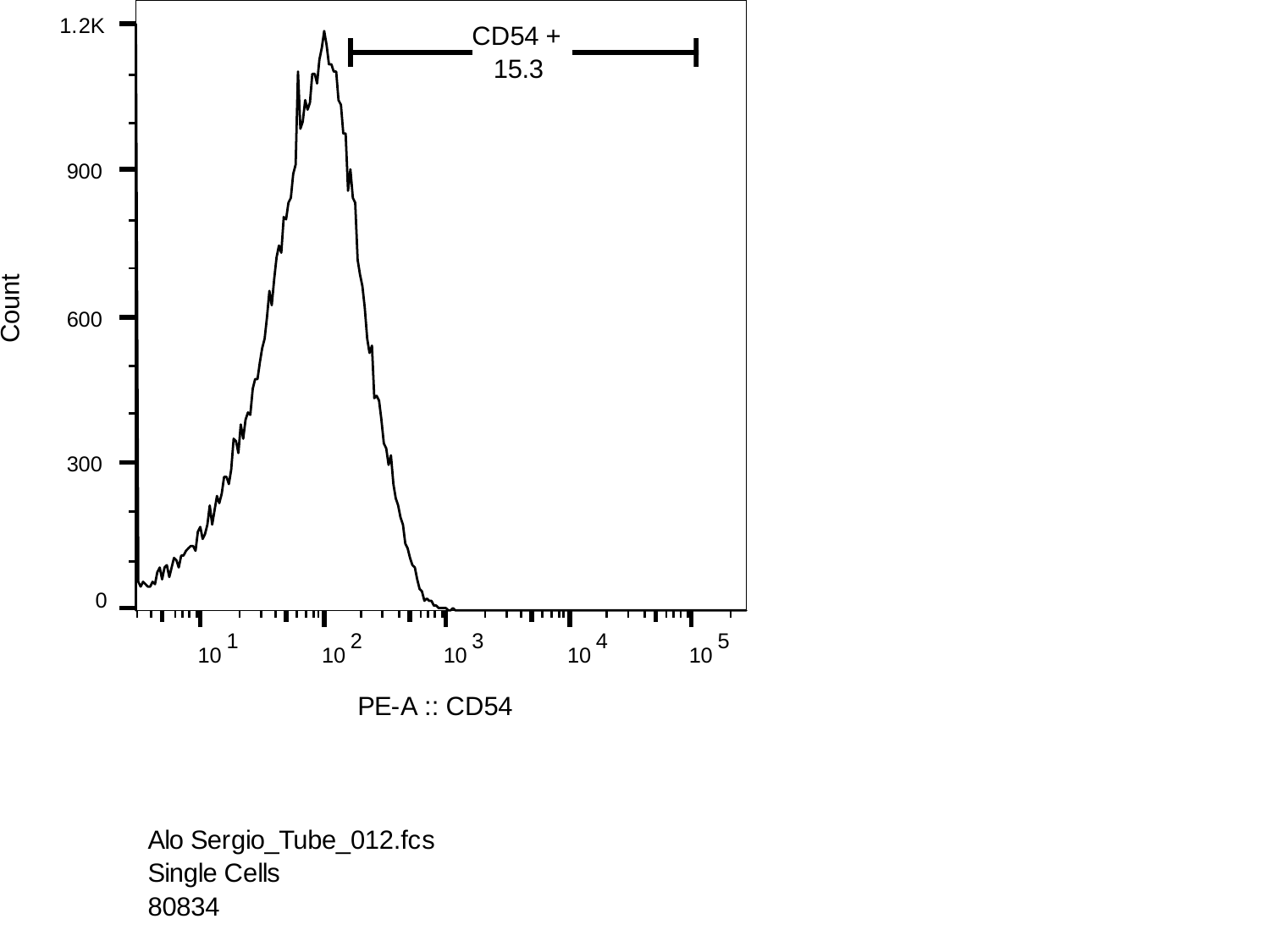

## Slide 14
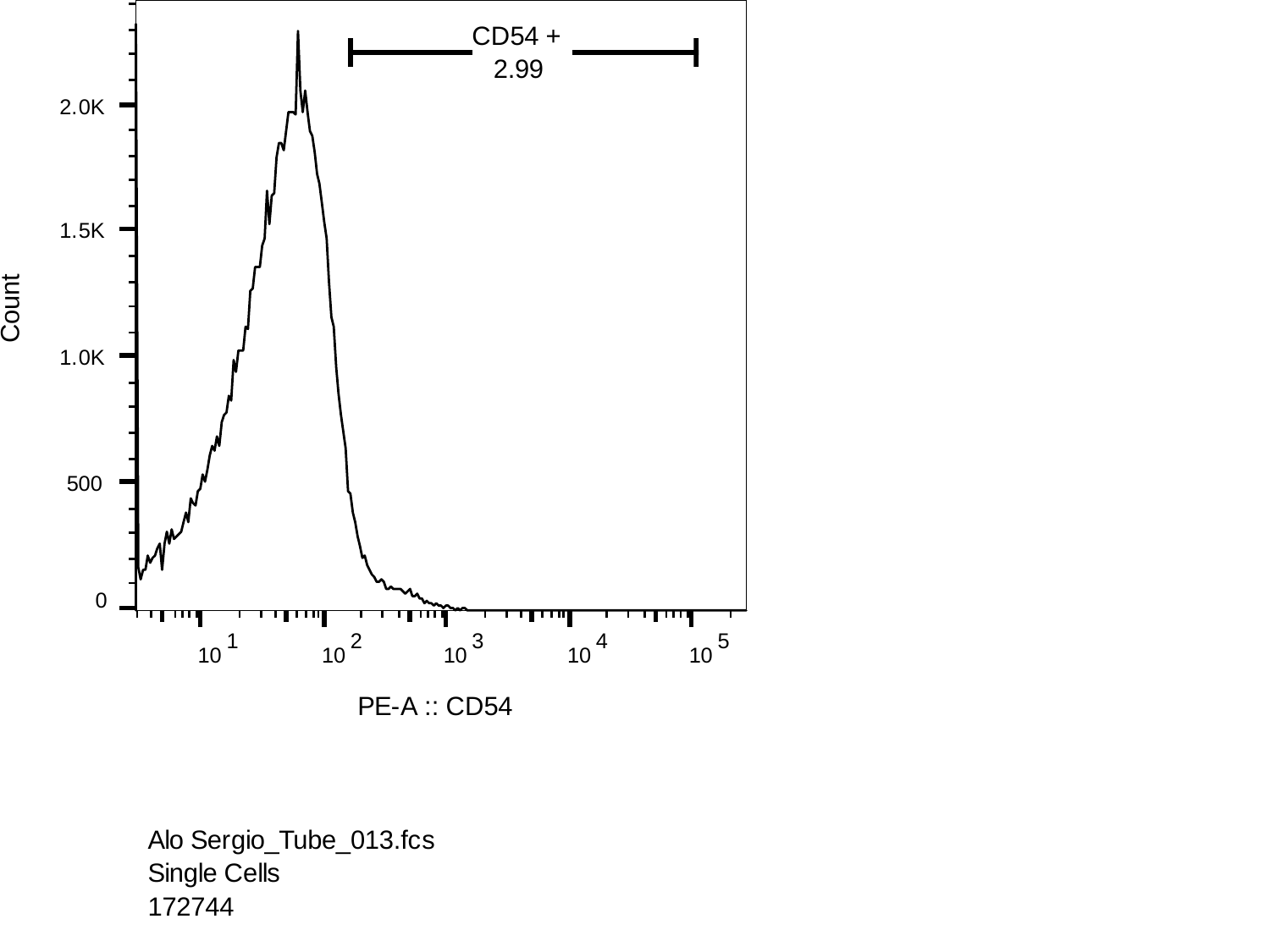

## Slide 15
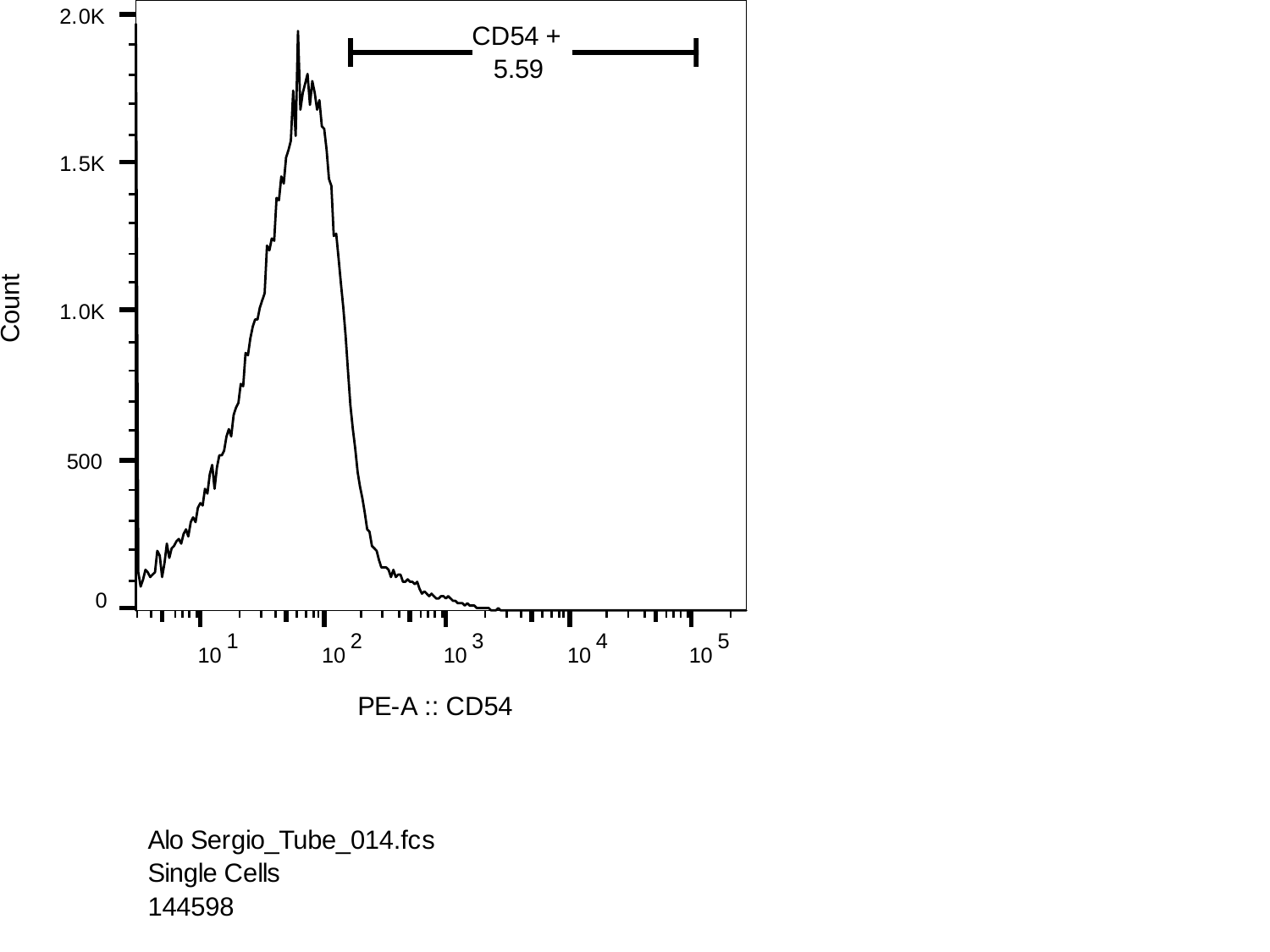

## Slide 16
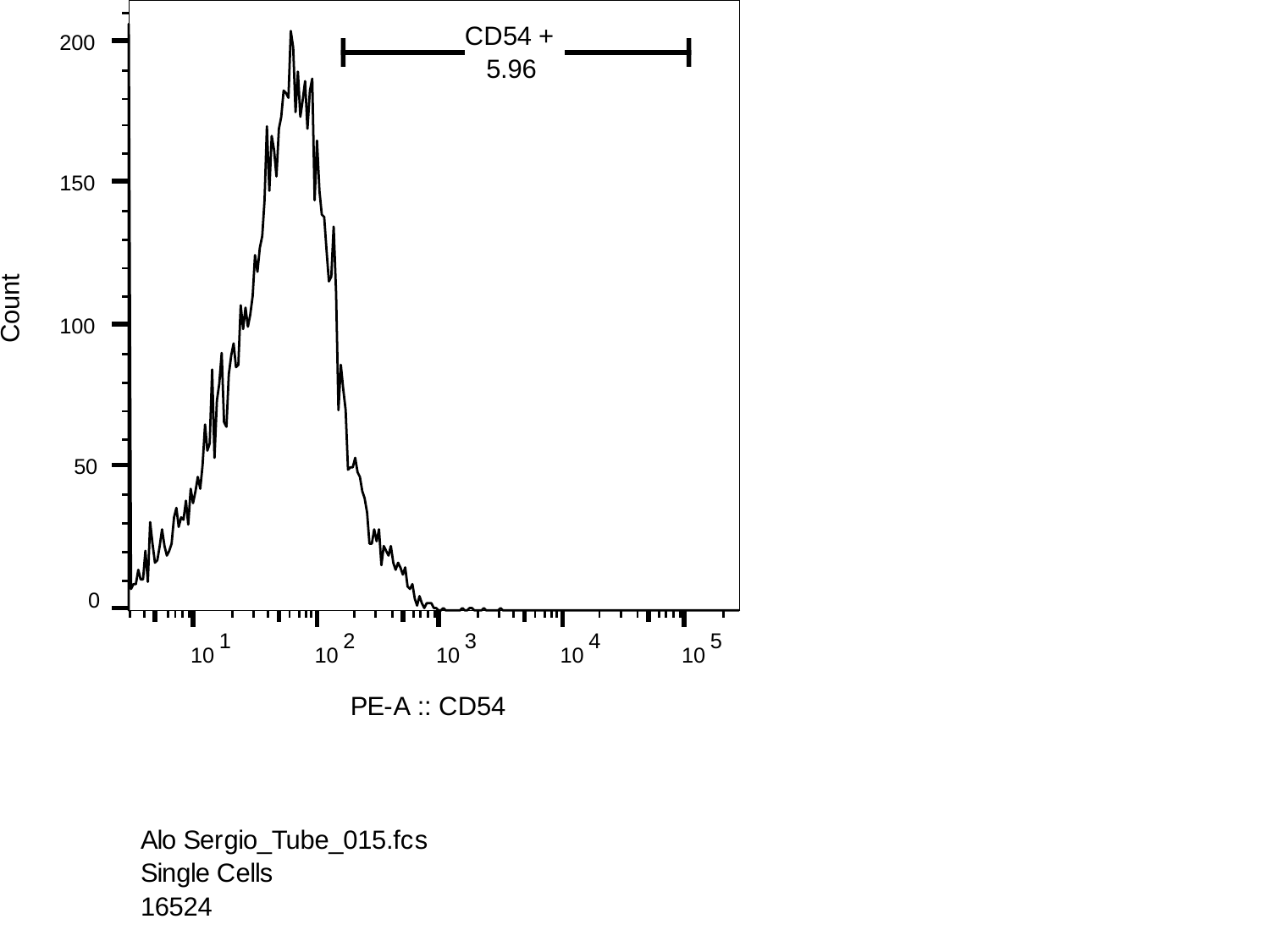

Supplement: Supplementary file 3 — Supplementary Material 3 [file 10103_2026_4883_MOESM3_ESM.pptx]
